# Locoregional Radiotherapy in Patients with Advanced Breast Cancer Treated with Cyclin-Dependent Kinase 4/6 Inhibitors Based on Real-World Data

**DOI:** 10.3390/ph17070927

**Published:** 2024-07-11

**Authors:** Marcin Kubeczko, Dorota Gabryś, Anna Polakiewicz-Gilowska, Barbara Bobek-Billewicz, Michał Jarząb

**Affiliations:** 1 Breast Cancer Center, Maria Sklodowska-Curie National Research Institute of Oncology, Gliwice Branch, 44-102 Gliwice, Poland; marcin.kubeczko@gliwice.nio.gov.pl (M.K.); anna.polakiewicz-gilowska@gliwice.nio.gov.pl (A.P.-G.); michal.jarzab@gliwice.nio.gov.pl (M.J.); 2 Department of Radiotherapy, Maria Skłodowska-Curie National Research Institute of Oncology, Gliwice Branch, 44-102 Gliwice, Poland; 3 Radiology and Diagnostic Imaging Department, Maria Skłodowska-Curie National Research Institute of Oncology, 44-102 Gliwice, Poland

**Keywords:** advanced breast cancer, cyclin-dependent kinase 4/6 inhibitors, radiotherapy

## Abstract

Background. The use of locoregional radiotherapy (RT) in patients with advanced ER-positive, HER2-negative breast cancer remains a topic of ongoing debate. In this study, we aimed to evaluate the efficacy of locoregional RT in advanced breast cancer patients treated with cyclin-dependent kinase 4/6 inhibitors (CDK4/6i) in a first-line setting. Methods. We conducted a retrospective analysis of patients diagnosed with advanced breast cancer between 2018 and 2023 who received treatment with CDK4/6i and underwent locoregional radiotherapy. Results. Among the 371 patients treated with CDK4/6i as part of their first-line therapy, 23 received locoregional RT either concurrently or sequentially with CDK4/6 inhibitors. Disease progression within the breast occurred in 19 patients (5.1%). Among these cases, five patients had previously undergone breast RT (5/23, 21.7%), while 14 did not (14/348, 4.0%, *p* = 0.004). All cases of local progression after RT followed palliative doses and were accompanied by early systemic progression. The 2-year PFS in the entire cohort of patients treated with locoregional RT was 65.7% (95% CI: 40.5–82.3%). Notably, patients who received higher RT doses had longer 2-year PFS (83.3%, 95% CI: 27.3–97.5%) than those with palliative RT doses (59.3%, 95% CI: 30.7–79.3%); however, the results were not statistically significant (*p* = 0.58). Furthermore, the 2-year local control in the entire cohort with locoregional RT was 73.0% (95% CI: 46.5–87.9%). Importantly, no local progression was observed after RT when using high doses. Conclusions. The addition of locoregional radiotherapy to first-line CDK4/6 inhibitors warrants further investigation across various clinical scenarios in advanced breast cancer. Palliative radiation regimens delivered early in breast oligoprogression may not always suffice, emphasizing the need for comprehensive studies in this context.

## 1. Introduction

In recent decades, there has been an increasing trend in breast cancer survival rates [1]. Although it is unclear whether patients at the metastatic stage can be cured, better systemic treatments have resulted in longer overall survival (OS) [2].

Patients with advanced HR-positive, HER2-negative breast cancer who received cyclin-dependent kinase 4/6 inhibitors (CDK4/6i) as a first-line treatment experienced extended progression-free survival compared to those treated in subsequent lines [3]. Furthermore, population-level enhancements in OS have been demonstrated for de novo metastatic breast cancer (MBC); however, these improvements have not been consistently observed in recurrent MBC cohorts or older women [4]. It is important to note that de novo metastatic breast cancer is heterogeneous and differs from recurrent disease. In the MONALEESA-2 trial, which evaluated combinations of ribociclib with letrozole as a first-line treatment for advanced breast cancer (ABC), patients with newly diagnosed metastatic disease experienced remarkably profound OS benefits [5].

For patients with HR+/HER2− ABC, current guidelines from the National Comprehensive Cancer Network and the European Society for Medical Oncology recommend CDK4/6i in combination with endocrine therapy as a preferred first-line treatment [6,7]. While systemic therapy remains crucial for treating metastatic breast cancer, local management of the primary breast tumor serves several purposes. Local treatment of the primary tumor can help alleviate symptoms such as pain, bleeding, or ulceration. However, it is essential to note that available data on the survival benefits of local treatment are limited [8,9,10,11]. Four prospective randomized trials investigated the role of surgery for primary tumors in a metastatic setting, involving 970 patients with de novo stage IV breast cancer [8,9,10,11]. Among these trials, only one reported an OS benefit [8]. However, quality of life outcomes showed no difference or deterioration after surgery in another trial [11]. Based on these findings, surgical intervention is generally not recommended for most metastatic patients unless specifically aimed at palliation and symptom management.

Quality of life is an essential factor in guiding treatment decisions. In most clinical trials involving CDK4/6i, quality of life was maintained or improved compared to endocrine therapy alone [12]. Furthermore, disease progression is associated with statistically significant and clinically relevant deterioration in various health-related quality-of-life parameters [13]. Therefore, prolonging progression-free survival remains an important treatment objective.

Radiotherapy for symptomatic breast tumors can provide durable palliation and reduce pain and bleeding [14]. Nonetheless, the use of locoregional radiation therapy in patients with advanced ER-positive, HER2-negative breast cancer remains a topic of ongoing debate.

In this study, we aimed to evaluate the efficacy and accuracy of locoregional RT in advanced breast cancer patients treated with CDK4/6 inhibitors in a first-line setting.

## 2. Results

### 2.1. Baseline Patient Characteristics

Three hundred and seventy-one patients were treated with CDK4/6i as part of their first-line therapy. Among them, 23 patients received locoregional radiotherapy (RT) and were included in further analysis. The median age was 57 years (range 32–80). Eighteen patients were diagnosed with de novo disease (78.3%), nine patients (39.1%) had metastases limited to bone, nine (39.1%) patients had both bone and visceral metastases, and two patients (8.7%) had visceral metastases without bone metastases (in the liver for one patient and in the lung for another patient). Two patients (8.7%) had only distant lymph node metastases (one to mediastinal lymph nodes and one to cervical lymph nodes). One patient (4.4%) had locally advanced inoperable BC without distant metastases. Thirteen patients were treated with ribociclib, seven with abemaciclib, and three with palbociclib. Twenty-one patients received aromatase inhibitors, whereas two patients received fulvestrant. The median follow-up was 22.4 months (IQR 14.6–39.8), and three patients (13.0%) were lost to follow-up.

### 2.2. Radiation Therapy

Fifteen patients received a palliative RT regimen (either 20 Gy delivered in 5 fractions or 30 Gy delivered in 10 fractions), and eight patients received higher doses of RT (26 Gy in 5 fractions, 42.5 Gy in 17 fractions, 45 Gy in 20 fractions, 50 Gy in 25 fractions, or 70 Gy in 30 fractions). Sixteen patients were treated concomitantly with CDK4/6i and seven patients underwent radiotherapy before CDK4/6i commencement.

The main reason for RT was oligoprogression within the breast (ten patients). Four patients received RT due to bleeding from breast ulceration and one due to pain. In six patients, locoregional RT was administered in an oligometastatic setting, while in two patients, metastatic disease was diagnosed during adjuvant radiation.

### 2.3. Treatment Efficacy

Among the 371 patients treated with CDK4/6i as part of their first-line therapy, disease progression within the breast occurred in 19 patients (5.1%). Among these cases, five patients had previously undergone breast RT (5/23, 21.7%), while 14 did not (14/348, 4.0%, *p* = 0.004). All cases of local progression followed RT with palliative doses and were accompanied by early systemic progression, usually in the visceral organs. Characteristics of patients with local progression after locoregional radiotherapy are shown in Table 1, while those with local control are shown in Table 2. In three of the four patients whose breast tumors were irradiated due to bleeding ulcerations, the bleeding was alleviated. Early adverse events from radiation therapy were limited and primarily represented by grade 1 and grade 2 reactions. Among the patients, eight experienced G1 skin toxicity, while three had G2 skin toxicity, as per The European Organization for Research and Treatment of Cancer (EORTC) criteria. No cases of severe late toxicity were reported.

### 2.4. Progression-Free Survival and Overall Survival

**Figure 1:**
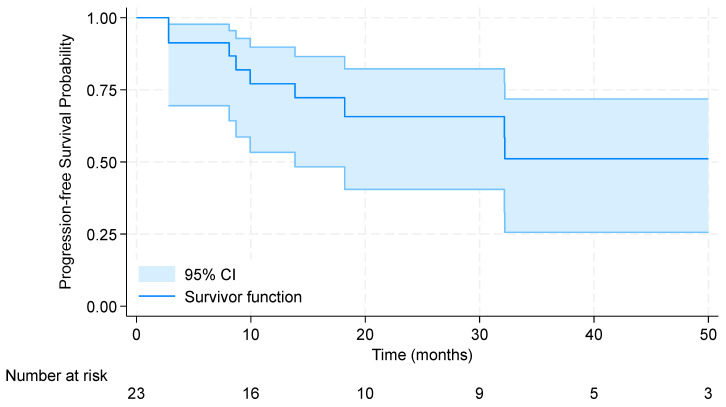
Kaplan–Meier curve for progression-free survival in the entire cohort of patients treated with locoregional radiotherapy.

**Figure 2:**
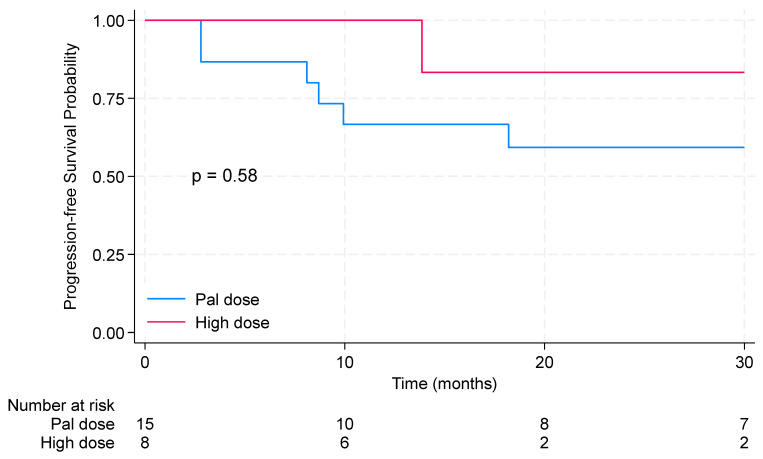
Kaplan–Meier curves for progression-free survival with comparisons between patients receiving high and palliative doses of locoregional radiotherapy. Pal dose: 20 Gy delivered in 5 fractions, or 30 Gy delivered in 10 fractions. Rad dose: 26 Gy in 5 fractions, 42.5 Gy in 17 fractions, 45 Gy in 20 fractions, 50 Gy in 25 fractions, or 70 Gy in 30 fractions.

The 2-year progression-free survival (PFS) in the entire cohort of patients treated with locoregional radiotherapy (RT) was 65.7% (95% CI: 40.5–82.3%). The median PFS was not reached. Results are shown in Figure 1. Notably, patients who received high RT doses demonstrated longer 2-year PFS (83.3%, 95% CI: 27.3–97.5%) than those with palliative RT regimens (59.3%, 95% CI: 30.7–79.3%); however, the results were not significant (*p* = 0.58). Results are displayed in Figure 2. The median overall survival for patients receiving locoregional radiation was 59.6 months. The 3-year OS was 70.4% [95% CI: 41.1–87.1%].

### 2.5. Local Control

**Figure 3:**
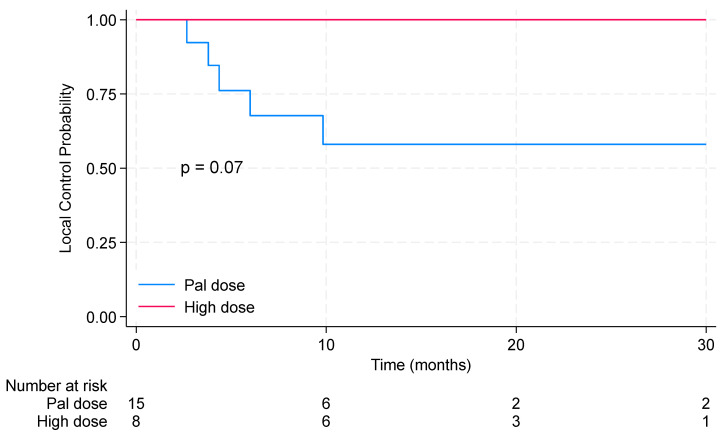
Kaplan–Meier curves for local control with comparisons between patients receiving high and palliative doses of locoregional radiotherapy. Pal dose: 20 Gy delivered in 5 fractions or 30 Gy delivered in 10 fractions. Rad dose: 26 Gy in 5 fractions, 42.5 Gy in 17 fractions, 45 Gy in 20 fractions, 50 Gy in 25 fractions, or 70 Gy in 30 fractions.

The 2-year local control in the entire cohort with locoregional RT was 73.0% (95% CI: 46.5–87.9%). The median local control was not reached. Importantly, no local progression was observed after RT when using high doses. Results are shown in Figure 3. Table 1 shows the characteristics of patients who did experience local progression, while those with local control are shown in Table 2.

#### 2.5.1. Time to In-Breast Disease Progression

The median time to in-breast progression was 59.6 months in patients with locoregional radiotherapy compared to 27.6 months in patients without locoregional radiotherapy (*p* = 0.061).

#### 2.5.2. Locoregional Radiotherapy in an Oligometastatic Setting

Among the treatment cohort, patients receiving locoregional radiotherapy (RT) in the oligometastatic setting constituted a minority (six patients, 26.1%). Although 2-year progression-free survival (PFS) numerically favored this subgroup (80.0%, 95% CI: 20.4–96.9%) compared to the remaining patients (62.4%, 95% CI: 34.5–81.2%), no statistically significant differences were observed (*p* = 0.40). Similarly, there were no significant differences in overall survival (OS) between these two subgroups (*p* = 0.64), nor in local control (LC) (*p* = 0.14).

#### 2.5.3. Comparison to Patients without Locoregional Radiotherapy

Our comparative analysis revealed no statistically significant differences between patients who received locoregional radiotherapy (RT) and those who did not across several parameters. Specifically, there was no notable difference in age (median age of 57, IQR 49–67 for RT patients vs. median age of 63.5, IQR 52–70 in patients who did not receive this treatment; *p* = 0.113), liver involvement (7, 30.4% vs. 64, 18.4%; *p* = 0.171) or lung involvement (6, 26.1% vs. 106, 30.5%, *p* = 0.816), nor performance status as per the Eastern Cooperative Oncology Group (ECOG 0: 12 patients, 52.2% in LR RT and ECOG 1 or 2: 11 patients, 47.8% in RT group compared to ECOG 0: 143 patients, 41.0% and ECOG 1 or 2: 205 patients, 58.9% in no RT group; *p* = 0.383). There were no significant differences in the cyclin-dependent kinase 4/6 inhibitor (palbociclib, ribociclib, or abemaciclib, *p* = 0.089) or endocrine compound (aromatase inhibitor vs. fulvestrant, *p* = 0.187) used. However, more patients in the locoregional group had de novo metastatic disease (18, 78.3% vs. 146, 42.0%, *p* = 0.001).

The median PFS among patients who received locoregional radiotherapy was not reached, contrasting a median PFS of 30.0 months observed in patients who did not receive this treatment. The 2-year PFS for patients treated with locoregional radiotherapy was 65.7% (95% CI: 40.5–82.3%) compared to 56.9% (95% CI: 50.6–62.8%) for patients without locoregional radiotherapy. However, the difference was not statistically significant (*p* = 0.321). Furthermore, the median overall survival for patients with locoregional radiotherapy was 59.6 months compared to 49.7 months for patients without locoregional radiotherapy. The 4-year OS in patients with locoregional radiotherapy was 70.4% (95% CI: 41.1–87.1%) compared to 55.4% (95% CI: 46.2–63.6%) for patients without locoregional radiotherapy. Nonetheless, the difference was not statistically significant (*p* = 0.795).

## 3. Discussion

High-quality randomized clinical trials have demonstrated that adjuvant breast radiotherapy provides clear benefits in terms of local control and survival for patients with early breast cancer [15]. Conversely, in a metastatic setting, the role of local therapy beyond palliation is undefined [16]. Thus, we performed a retrospective study to assess the role of locoregional RT in patients diagnosed with advanced breast cancer who undergo CDK4/6 inhibitor treatment in a first-line setting.

The addition of CDK4/6i to endocrine treatment resulted in a median PFS in PALOMA-2 of 24.8 months [17], whereas in the MONARCH-3 study, the median PFS was 28.2 months [18]. In the MONALEESA project, the median PFS was 25.3 months [19], 20.5 months [20], and 23.8 months [21] for MONALEESA-2, -3, and -7, respectively. Given these results, a 2-year PFS was selected as the primary study outcome. In terms of overall survival, the median OS in the PALOMA-2 study for the CDK4/6i and endocrine treatment arm was 53.9. In the MONALEESA project, the median OS was 53.7 months [3], 67.6 months [22], and 58.7 months [23] for MONALEESA-2, -3, and -7, respectively. Accordingly, a 4-year OS was established as a secondary study outcome.

Pivotal randomized trials did not yield sufficient information regarding the safety and efficacy of palliative locoregional therapy. In the context of ribociclib treatment assessed in MONALEESA studies, only radiotherapy with palliative regimens for bone pain was feasible [5,21,24]. During the MONARCH trials, which evaluated abemaciclib’s efficacy, radiotherapy was not permitted [2,18]. Regarding palbociclib therapy, palliative radiotherapy was only allowed in the PALOMA trials [17,25]. In the context of limited data, studies such as ours have the potential to provide crucial insights for a significant number of patients in their daily medical care. Radiotherapy administered with palliative regimens was prescribed to most patients in our study and demonstrated some efficacy, even within an oligoprogressive setting.

There is also a shortage of data on concurrent adjuvant locoregional radiotherapy and CDK4/6i treatment from randomized trials [26,27,28]. Thus, the European Society for Radiotherapy and Oncology-endorsed recommendations suggest caution when combining CDK4/6i with adjuvant radiotherapy [29]. Recently published meta-analyses regarding CDK4/6i and radiotherapy combinations did not reveal enhanced severe toxicity [30,31]. Furthermore, in a recent retrospective analysis, concurrent administration of locoregional RT and CDK4/6 inhibitors did not cause severe late toxicities in most patients [32]. In our study, most patients underwent locoregional radiotherapy at higher doses, either sequentially to CDK4/6 inhibitors or with CDK4/6 inhibitor interruption. This treatment approach demonstrated remarkable efficacy in a carefully selected subset of patients.

Most studies examining locoregional treatment primarily concentrate on patients with de novo disease [16,32,33]. In our study, patients with de novo disease constituted nearly 80% of the study cohort. However, we decided to analyze all patients who received CDK4/6 inhibitors in the first-line setting. By considering both de novo and recurrent cases, we tried to capture a more comprehensive picture of the patient population. Additionally, this approach is particularly relevant for those who did not receive previous radiotherapy after a mastectomy. This inclusion allows for a more nuanced evaluation and potentially identifies candidates who could benefit from locoregional radiotherapy at higher doses in the recurrent setting. Moreover, the emerging role of partial breast irradiation in the early disease setting introduces the potential for reirradiation in recurrent cases [34].

When considering the treatment of primary tumors in metastatic disease, several potential mechanisms may offer benefits [35,36]. Additionally, in the context of patients with hormone receptor-positive breast cancer, ribociclib had a significant impact on both the peripheral innate and adaptive immune response [37]. Thus, additional mechanisms may play a role in combining radiotherapy and CDK4/6i benefits.

Several retrospective studies have explored the combination of CDK4/6 inhibitors and radiotherapy [38,39,40,41,42,43,44,45,46,47,48,49,50,51,52,53]. Nonetheless, available data concerning the population of patients receiving locoregional treatment remain relatively limited.

In our study, a substantial cohort of patients received systemic treatment following a similar approach. On the other hand, the reasons for local treatment vary substantially, from palliation for ulcerative disease with bleeding to oligometastatic disease treatment. We are fully aware of entirely different prognoses for such different clinical scenarios. The median OS for stage IV breast cancer patients varies widely by patient factors such as age, performance status, and comorbidities; cancer-related factors, including tumor burden, location of metastases, and biological subtype; and treatment factors, such as accessibility to efficient systemic treatment [54]. Our findings highlight the diverse approaches taken to managing advanced breast cancer patients.

Radiation therapy for stage IV breast cancer encompasses a spectrum of clinical scenarios. A retrospective analysis of data from 27 patients with de novo metastatic breast cancer treated with CDK4/6 inhibitors and locoregional irradiation was recently published [32]. The study reported a 2-year PFS of 53.7% [95% CI: 35.8–80.5%], which is comparable to outcomes observed in our cohort with a 2-year PFS of 65.7% (95% CI: 40.5–82.3%).

However, the bulk of research in this domain predates the advent of targeted therapies such as CDK4/6i [55], with systemic treatment paradigms evolving substantially since then. This evolution has introduced complexities in interpreting past studies’ relevance to current practice. Moreover, the literature presents notable discrepancies. For instance, a large meta-analysis involving stage IV breast cancer patients who underwent breast surgery between 2010 and 2015 highlighted the mortality reduction potential of postoperative radiotherapy, qualifying at 31.8% [56]. In stark contrast, another meta-analysis concluded that locoregional therapy in de novo metastatic disease did not correlate with enhanced overall survival, irrespective of the presence of bone-only or visceral metastases or the tumor subtype [16].

Studies investigating the role of locoregional radiotherapy in primary breast tumors during the era of modern systemic treatment, which has evolved dramatically over the last decade, are lacking [57]. The optimal integration of locoregional treatment with modern systemic treatment remains unknown. The question at hand is whether highly effective systemic treatments complement aggressive strategies with locoregional therapy to eradicate breast cancer, or if they potentially undermine the advantages of locoregional therapy, thus nullifying its ability to enhance survival benefits [57]. Although both scenarios may hold validity, their applicability may vary among distinct patient populations. This idea suggests a nuanced interplay where the efficacy of combining systemic and locoregional treatments depends on individual patient characteristics and disease profiles.

The therapeutic landscape for hormone receptor-positive human epidermal growth factor receptor 2-negative metastatic breast cancer is rapidly evolving, with treatments tailored to specific mutations or histopathologic features. The following therapies were recently approved for this breast cancer subtype: elacestrant [58] (in estrogen receptor 1-mutated breast cancer) aleplisib [59,60] (addressing PIK3CA mutations) and poly(adenosine diphosphate-ribose) polymerase (PARP) inhibitors, such as talazoparib [61] or olaparib [62] (for patients with germline pathogenic variants of BRCA1 or BRCA2 genes). Emerging treatments poised to enhance clinical practice include camizestrant [63] and inavolisib [64]. Antibody–drug conjugates, such as trastuzumab deruxtecan [65] and sacituzumab govitecan [66] have also proved effective in this specific subtype. For the HER2-positive subtype and triple-negative breast cancer, recent approvals have expanded options with drugs such as tucatinib [67], trastuzumab deruxtecan [68], pembrolizumab [69], and sacituzumab govitecan [70].

These advancements underscore the shift away from a one-size-fits-all approach, particularly in the context of locoregional radiotherapy in a metastatic setting. The benefits observed extend beyond progression-free survival to overall survival, highlighting the need for personalized treatment strategies. Our studies are crucial in bridging the knowledge gap in these swiftly changing clinical scenarios.

We acknowledge the modest size of our patient cohort undergoing locoregional radiotherapy, which inherently constrains our findings’ broad applicability. Nevertheless, recognizing the substantial unmet medical needs within this subgroup is imperative. The pronounced gap between existing clinical practices and supportive research data is considerable. In light of this, our investigation holds significant value in substantiating current clinical approaches. It serves as a critical interim resource while we anticipate forthcoming data from prospective clinical trials that encompass a more extensive patient population.

Further research and evaluation are essential to optimizing treatment strategies. Currently, we await the results of prospective studies. The PALATINE trial (NCT03870919) aims to investigate the integration of locoregional treatment with palbociclib in patients with de novo, treatment-naïve, stage IV ER-positive, and HER2-negative breast cancer.

We recognize that our study did not yield statistically significant results. For the majority of patients, the main problem is disease progression within the visceral organs, predominantly affecting the liver and lungs. This progression complicates the demonstration of a progression-free survival advantage when incorporating locoregional radiotherapy into the treatment regimen. Despite this, we observed an encouraging trend: patients receiving higher radiation doses tended toward improved local control. Although these findings were not statistically significant, it is important to note that this may be attributed to the relatively small patient cohort.

Among the treatment cohort, patients receiving locoregional radiotherapy in an oligometastatic setting constituted a minority. Consequently, we could not draw specific conclusions about that particular population. Larger prospective studies are necessary to address this gap, considering the distinct biology of these cancers and their different progression patterns.

Progression-free survival and overall survival remain crucial endpoints in studies of metastatic breast cancer. However, identifying patients at risk of local tumor progression in the breast or axilla is essential. Radiotherapy may offer significant benefits in such cases, improving quality of life and beyond.

For now, the decision to add locoregional radiotherapy to CDK4/6 inhibitor treatment warrants thoughtful consideration. Balancing the potential benefits with the impact on patients’ well-being is crucial.

## 4. Material and Methods

### 4.1. Study Population and Therapy Details

Patients diagnosed with advanced breast cancer who received treatment with Cyclin-dependent kinase 4/6 inhibitors (CDK4/6i) and locoregional radiation therapy between 2018 and 2023 were retrospectively analyzed.

Patients regularly visited our cancer center, including the Breast Cancer Center and the Department of Radiotherapy. A comprehensive database was assembled from our institution’s electronic health records. The data collected encompassed a wide range of clinical factors, including patient age, disease status with differentiation between de novo metastatic and recurrent disease status, histology, performance status according to the Eastern Cooperative Oncology Group, metastatic sites; baseline visceral organ involvement, including liver and lung; line of treatment, a cyclin-dependent kinase 4/6 inhibitor type, endocrine compound, progression-free survival, overall survival, radiotherapy dosage: total dose and dose per fraction delivered with radiotherapy, radiation volume, radiotherapy equipment, and post-radiotherapy local control.

Inclusion criteria were as follows: (1) patients diagnosed with advanced breast cancer; (2) hormone receptor-positive human epidermal growth factor receptor 2-negative subtype; (3) targeted therapy—patients undergoing treatment with a cyclin-dependent kinase 4/6 inhibitor (specifically one of the following: palbociclib, ribociclib, or abemaciclib); (4) concurrent treatment with endocrine therapy (such as an aromatase inhibitor or fulvestrant); (5) radiation therapy to the breast/regional lymph nodes performed concurrently with CDK4/6i or up to 6 months prior to the commencement of CDK4/6i.

Exclusion criteria: (1) patients diagnosed with early-stage breast cancer; (2) those with triple-negative breast cancer or human epidermal growth factor receptor 2-positive subtype; (3) patients who were not receiving any cyclin-dependent kinase 4/6 inhibitor treatment; (4) patients who were receiving a cyclin-dependent kinase 4/6 inhibitor in combination with treatments other than endocrine therapy.

CDK4/6i, namely palbociclib, ribociclib, and abemaciclib, were administered according to the prescribed information. Almost all radiation treatments were performed using a Linear Accelerator (Varian a Siemens Healthineers Company, Palo Alto, CA, USA), except for one performed with CyberKnife (Accuray, Sunnyvale, CA, USA). The multidisciplinary team made decisions regarding systemic treatment and radiotherapy.

Palliative regimens comprised 20 Gy delivered in 5 fractions and 30 Gy delivered in 10 fractions. High-dose RT regimens comprised 42.5 Gy in 17 fractions, 45 Gy in 20 fractions, 50 Gy in 25 fractions, 70 Gy in 30, and 26 Gy in 5 fractions.

The primary study outcome was 2-year progression-free survival in patients receiving locoregional radiotherapy during CDK4/6i first-line treatment of advanced breast cancer. The secondary outcomes were 2-year local control in patients receiving locoregional RT and 4-year overall survival.

Time to in-breast progression was established as an additional secondary outcome. It was defined as the time from CDK4/6i commencement to disease progression within the breast region. This analysis encompasses patients who have undergone locoregional radiotherapy as well as those who have experienced disease progression, specifically in the breast region.

Response Evaluation Criteria in Solid Tumours version 1.1 was used for response assessments based on the following categories: complete response, partial response, progressive disease, or stable disease [71].

Progression-free survival was calculated from the date of beginning CDK4/6i to the date of disease progression according to RECIST 1.1 or death. Overall survival was calculated from the date of beginning CDK4/6i to the date of death. Local control was calculated from the end of radiotherapy to local progression or the last visit. If the patient was lost to follow-up or died before diagnosis of local progression, LC status was censored at the time of the last visit.

All data were derived from real-life settings without additional visits associated with the study.

### 4.2. Statistical Analysis

Categorical variables were shown as frequencies and percentages. Continuous data were displayed with median values and the interquartile range (25% to 75%, IQR). Fisher’s exact test assessed differences between categorical variables. Progression-free survival, overall survival, and local control were estimated using the Kaplan–Meier method, with a log-rank test evaluation for the differences. For the survival curves, 95% confidence intervals (CIs) were calculated. All tests were two-sided. A *p*-value ≤ 0.05 indicated statistical significance. Stata Statistical software (version 18, StataCorp, College Station, TX, USA) was used for all analyses.

## 5. Conclusions

The addition of locoregional radiotherapy to first-line CDK4/6 inhibitors warrants further investigation across various clinical scenarios in advanced breast cancer. Palliative radiation regimens administered to patients with early oligoprogression may not always suffice, emphasizing the need for comprehensive studies in this context.

## Figures and Tables

**Table 1:** Characteristics of patients with local progression after locoregional radiotherapy.

cTNM	RTd	RT Site	RT T	LC (mth)	PFS (mth)	PD Site	OS (mth)
RT due to breast oligoprogression							
De novo T4bN1M1	20/4	Breast	D	4.0	8.2	breast, axilla, lung	14.6 +
Rc w/o prev. RT	30/3	Chest wall and LN	D	2.7	9.9	Chest wall, lung	19.6 #
Rc w/o prev. RT	20/4	Chest wall	D	6.0	18.2	Chest wall, lung	26.9 +
RT due to symptoms (bleeding from breast tumor ulceration or pain)							
De novo T4bN3M1	20/4	Breast and LN	B	3.8	2.8	Breast, lung, liver	11.2 +
De novo T4bN3M1	20/4	Breast and LN	D	7.1	8.1	Breast	15.8 #

Abbreviations: RT—radiotherapy; RTd—dose of RT, total dose/dose per fraction; RT T – RT timing; D—during cyclin-dependent kinase 4/6 inhibitor treatment; B – before cyclin-dependent kinase 4/6 inhibitor treatment; LC—local control; mth—months; PFS—progression-free survival; PD—disease progression; OS—overall survival; +—patient died; #—patient lost to follow-up; Rc – recurrent; w/o—without; b.—before the commencement of cyclin-dependent kinase 4/6 inhibitors; prev.—previous.

**Table 2:** Characteristics of patients with local control after locoregional radiotherapy.

cTNM	RTd	RT Site	RT T	LC (mth)	PFS (mth)	PD Site	OS (mth)
RT due to breast oligoprogression							
Rc	26/5.2	Second Breast	D	16.7	32.2	Liver	51.2
De novo T2N0M1	30/3	Breast	D	10.9	34.6	Cont CDK	35.7
De novo T4cN3M1	30/3	Breast and chest wall	D	6.6	32.2	second breast	36.6
De novo T4bN1M1	20/4	Breast	D	1.2	23.6	Cont CDK	26.2
Rc after APBI rcT4(b)Nx	20/4	Breast	D	11.0	39.8	Cont CDK	42.6
De novo T4cN1M1	30/3	Breast	D	5.7	52.5	Lost to FU	62.2 +
De novo T4cN3M1	20/4	Breast and LN	D	1.4	40.3	Cont CDK	42.7
RT due to bleeding from breast tumor ulceration							
De novo T4bN2M1	30/3	Breast and LN	D	35.8	40.9	Cont CDK	42.4
Rc w/o prev RT	20/4	Breast and chest wall	B	4.0	2.8	Lost to FU	4.2
De novo T4bN1M1	20/4	Breast	B	15.0	14.6	Cont CDK	16.0
RT in oligometastatic disease							
De novo T3N1M1	45/2.25	Chest wall and LN	B	65.9	59.9	Cont CDK	69.5
De novo T4bN1M1	42.5/2.5	Breast and LN	D	15.9	17.0	Cont CDK	31.1
De novo T4dN2M1	50/2	Breast and LN	D	16.7	17.2	Cont CDK	26.7
De novo T3(m)N1M1	70/2.33 *	Breast and LN	D	2.6	8.5	Cont CDK	11.2
De novo T4bN1M1	42.5/2.5	Chest wall and LN	B	24.4	16.9	Cont CDK	33.8
De novo T2N3M1	50/2	Breast and LN	B	24.3	13.9	Liver	37.2 +
Metastatic disease diagnosed during adjuvant RT							
De novo T1N1M1	42.5/2.5	Breast and LN	B	5.3	5.1	Lost to FU	7.0 #
De novo T1N1M1	50/2 **	Breast and LN	B	64.3	63.3	Cont CDK	70.0

Abbreviations: RT—radiotherapy; RTd—dose of RT, total dose/dose per fraction; CDK—cyclin-dependent kinase 4/6 inhibitor; RT T—RT timing; D—during cyclin-dependent kinase 4/6 inhibitor treatment; B—before cyclin-dependent kinase 4/6 inhibitor treatment; LC—local control, mth—months; wk—weeks; PFS—progression-free survival; PD—disease progression; OS—overall survival; Rc—recurrent; +—patient died; #—patient lost to follow-up; w/o—without; b.—before the commencement of cyclin-dependent kinase 4/6 inhibitors; prev.—previous; LN—lymph nodes; * Breast and axillary LN and supraclavicular LN 50/1.66, breast and axillary LN 60/2, axillary LN 66/2.2, breast tumor 70/2.33; CDK4/6i was interrupted during this treatment. ** Adjuvant RT was stopped when metastatic disease diagnosis was confirmed, after delivering 10 Gy.

## Data Availability

Data is contained within the article.
